# Efficacy of Essential Oils from Edible Plants as Insecticides Against the House Fly, *Musca Domestica* L

**DOI:** 10.3390/molecules14051938

**Published:** 2009-05-25

**Authors:** Sara M. Palacios, Alberto Bertoni, Yanina Rossi, Rocío Santander, Alejandro Urzúa

**Affiliations:** 1 Laboratory of Fine Chemicals and Natural Products, Faculty of Chemical Sciences. Catholic University of Córdoba, Camino a Alta Gracia Km 10, (5000) Córdoba, Argentina. Fax: 0351-4938061. Tel.: 0351- 4938060; E-mails: bertonialberto@hotmal.com (A.B.); yanirro@hotmail.com (Y.R.); 2 Facultad de Química y Biología, Universidad de Santiago de Chile, Casilla 40, Correo 33, Santiago, Chile. Phone: 56-2-7181155; Fax: 56-2-6812108; E-mail: aurzuamoll44@yahoo.com (A.U.); rocio.santander@gmail.com (R.S.)

**Keywords:** *Musca domestica*, natural insecticide, essential oils, *Citrus sinensis*, *Eucalyptus cinerea*

## Abstract

The compositions of 12 essential oils (EOs) obtained by hydrodistillation of edible fruits and herbs were analyzed by gas chromatography/mass spectroscopy (GC/MS). The insecticidal activity of each oil against the house fly *Musca domestica* was evaluated by placing flies in a glass jar with a screw cap that held a piece of EO-treated cotton yarn. The dose necessary to kill 50% of flies (LC_50_) in 30 min was determined at 26 ± 1°C. Twelve EOs and 17 individual terpenes were assayed against *M. domestica*, showing LC_50_ values ranging from 3.9 to 85.2 and from 3.3 to >100 mg/dm^3^, respectively. EO from *Citrus sinensis* was the most potent insecticide (LC_50_ = 3.9 mg/dm^3^), followed by EOs from *C. aurantium* (LC_50_ = 4.8 mg/dm^3^) and *Eucalyptus cinerea* (LC_50_ = 5.5 mg/dm^3^). According to GC/MS analysis, limonene (92.47%), linalool (1.43%), and β-myrcene (0.88%) were the principal components of *C. sinensis* EO. Limonene was also the principal constituent (94.07%) of *C. aurantium*, while 1,8-cineole (56.86%) was the major constituent of *E. cinerea* EO. 1,8-Cineole was most active against *M. domestica* (LC_50_ = 3.3 mg/dm^3^), while (4*R*)(+)-limonene, was moderately active (LC_50_ = 6.2 mg/dm^3^). Dimethyl 2,2-dichlorovinyl phosphate (DDVP) selected as a positive control, showed an LC_50_ of 0.5 mg/dm^3^. EOs from *C. sinensis, C. aurantium*, and *E. cinerea* show promise as natural insecticides against houseflies.

## 1. Introduction

The housefly, *Musca domestica* L., is one of the most common insects, intimately associated with human settlements, food, and utensils. Flies feed and breed on decaying matter, human waste, and food, and are therefore considered to be mechanical vectors of pathogens such as bacteria, protozoa, and viruses. The housefly is categorized by the U.S. Food and Drug Administration as an important contributing factor in the dissemination of various infectious food-borne diseases such as cholera, shigellosis, and salmonellosis [1]. Adult houseflies have been shown to transmit pathogens from their sponging mouthparts, through vomitus, on their body and leg hairs, on the sticky parts of the feet, and through the intestinal tract, [2] thereby contaminating food and propagating disease.

Many insecticides such as organochlorines and organophosphates, and more recently pyrethroids and spinosad, have been used for housefly control. However, houseflies can develop resistance to these pesticides [3,4] and health and environmental risks are associated with these compounds; thus, investigators continue to search for alternative methods of fly management. In this sense, essential oils (EOs) and natural terpenes (Ts) are potential alternatives and environmental friendly insecticides [5,6,7].

The toxicity of Ts against *M. domestica* has been studied extensively by Coats *et al*. [8] who demonstrated their insecticidal activity against a variety of insects via topical application. Among 33 Ts tested topically on *M. domestica*, thymol and pulegone were found to be the most active; the concentrations required to kill 50% of the insects (LC_50_) were 29 and 39 μg/fly, respectively [9]. However, *d*-limonene was more active than pulegone (LC_50_ = 10 and 166 μg/fly, respectively) when applied topically to female houseflies [8]. In a study in which monoterpenoid ester derivatives were synthesized from their parent alcohol or phenol, the parent compound was generally the more active compound against *M. domestica* [10].

An investigation of topical versus fumigant toxicity for a group of Ts revealed different levels of effectiveness for the same terpene (T). For instance, the most toxic topically applied Ts were, in descending order, thymol, citronellic acid, and citronellal, with topical LC_50_s of 33, 43, and 60 μg/fly, respectively. Within the same group of Ts, the best fumigants were citronellal, menthol, and *l*‑fenchone, with fumigant LC_50_s of 2, 3.6, and 3.8 μg/cm^3^ [11]. The authors' explanation for the differences in activity of a given T was that fumigant toxicity is a function of terpene volatility. A bioassay on adult house flies using topical application of 1,8-cineole indicated that males were more susceptible than females, with LC_50_ values of 118 and 177 μg/fly, respectively [12]. Phenols like thymol, and carvacrol were more topically toxic than saturated alcohols, but saturated alcohols were better fumigants against houseflies than were phenols [11].

Extensive study on the insecticidal properties of Ts has demonstrated that a given T has different levels of activity in different insects [11,13] and that Ts belonging to the same chemical family seem to have different modes of action in the same insects [14]. 

Data on the topical and fumigant activity of EOs against *M. domestica* are scarce [15]. *Matricaria chamomilla* and *Clerodendron inerme* EOs have topical LC_50_s of 76 and 84 μg/fly, respectively [16]. EOs from *Citrus sinensis* and *C. aurantifolia* peel showed 70% fumigant effectiveness in killing house flies in a room after 60 min of spraying [17]. The insecticidal properties of lemon peel, grapefruit, and navel orange citrus oils against adults and larvae of *M*. *domestica* have been investigated [18], and grapefruit peel oil was toxic to *M. domestica* adults while lemon oil was toxic to larvae. *Piper betle* EOshowed a fumigant LC_50_ of 10.3 mg/dm^3^ in a 24-h exposure period [19]. More recently, 34 EOs were screened against the housefly, and *Pogostemon cablin* EO was found to be the most potent topical insecticide, with an LD_50_ of 3 μg/fly; *Mentha pulegium* oil was the most potent fumigant insecticide (LC_50_ = 4.7 μg/cm^3^) [20].

Many studies have evaluated of the use of Ts for controlling larvae and adult house flies in areas where this insect develops, but little has been done to evaluate Ts or EOs as fumigants in areas involved in food preparation or consumption or in domestic areas. Therefore, the literature offers little guidance as to the best choice of EO for use in a human environment. For instance, the reported LC_50_ values of EOs or Ts were determined at 24 h or more after topical application or after 24 h of exposure. In order to control flies that threaten food dishes, EOs must be effective within a shorter period. In addition, most previous studies did not report the bioassay temperature as being an important parameter with regard to volatile compounds. In this study, we evaluated the effectiveness of 12 EOs derived from edible plants and fruits against *M. domestica* after 30 min of exposure in order to select the best candidate for the formulation of new household products. 

## 2. Results and Discussion

The compositions of each of the 12 EOs were consistent with previously reported compositions; the main components are listed in Table 1. The fumigant effects of EOs against adult *M. domestica* were evaluated by determining the LC_50_ values, which are presented in Table 2. The most effective insecticide was the EO derived from *C. sinensis*, followed by *C. aurantium* and *E. cinerea*, with LC_50_ values of 3.9, 4.8, and 5.5 mg/dm^3^ of air respectively. EOs derived from other citrus species were also very effective against *M. domestica*, with LC_50_ values ranging from 6.5 to 7 mg/dm^3^. Some EOs were modestly to marginally effective, requiring doses from 8.8 to more than 25 mg/dm^3^ to induce 50% mortality. However, all EOs could kill some individual *M. domestica* adults within 30 minutes.

To determine whether contact toxicity contributed to the insecticidal properties of the various EOs, bioassays using of the most active EOs (sweet orange, sour orange, and eucalyptus) were conducted such that *M. domestica* could not come into contact with the cotton; similar mortality rates were observed (data not shown). This result was expected because contact and fumigant toxicity cannot be differentiated in cases of volatile compounds, since the compounds can reach any part of the fly body and our flies had full access to the entire test vessel.

**Table 1:** Components (percent) of the essential oil assayed.

	*RI*	*Cau*	*Cl*	*Cp*	*Cr*	*Cs*	*C sa*	*Ec*	*Ln*	*Mf*	*Mp*	*Pa*	*Sa*
α-Thujene	**924**									1.13			
α-Pinene	**932**		2.35		1.62		5.63	6.42	3.90	8.25			
Sabinene	**961**			1.21									
β-Pinene	**970**		9.59										
β-Myrcene	**983**			2.62	2.49	0.88			1.21	3.96			
α-Phellandrene	**1002**									1.52			
δ-3-Carene	**1008**									1.90			
α-Terpinene	**1014**									5.28			
Limonene^a^	**1027**	94.07	49.56	94.97	82.83	92.47							
m-Cymene	**1030**						4.34						
1,8-Cineol	**1035**							56.86	21.66		6.73		
δ-Terpinene	**1050**		10.18		10.89		6.55		1.40	7.14			
α-Terpinolene	**1082**		1.76							3.42			
Linalool	**1121**					1.43	63.56		11.90				
Camphor	**1137**						5.36						
Menthone	**1144**										20.88		
Mentol	**1164**										41.51		
Terpinen-4-ol	**1182**		3.17					1.24	3.36	13.69			
Terpineol	**1190**		6.35			0.28				1.82			
Estragole	**1195**											3.42	
Pulegone	**1234**										2.57		
*p*-Anisaldehyde	**1240**											6.31	
Geraniol	**1265**						2.86						
Safrol	**1275**									5.21			
*trans*-Anethole	**1282**											68.76	
Neryl acetate	**1359**		3.61										
Eugenol	**1366**								2.90				63.26
Geranyl acetate	**1379**		1.06				4.87						
Eugenol methyl	**1408**								8.10				
β-Caryophyllene	**1408**									1.00			22.62
α-Humulene	**1452**												3.43
α-Himachalene	**1490**											11.88	
β-Bisabolene	**1530**		1.25									1.25	
Eugenyl acetate	**1546**												6.28
Caryophyllene oxide	**1582**										1.20		

Cau, *Citrus aurantium*; Cl, *Citrus limon*; Cp, *Citrus paradise*; Cr, *Citrus reticulata*; Cs, *Citrus sinensis*; Csa, *Coriandrum sativum*; Ec, *Eucalyptus cinerea*; Ln, *Laurus nobilis*; Mf, *Myristica fragrans*; Mp, *Mentha piperita*; Pa, *Pimpinella anisum*; Sa, *Syzygium aromaticum*. a) (4*R*)(+)-limonene >99% by GC chiral columna, see experimental section.

**Table 2:** LC_50_s of essential oils against *Musca domestica.*

Essential oil	Mean LC_50_ in mg/dm^3^ (95% CI)
*Citrus aurantium*	4.8 (3.7 - 6.3)
*Citrus limon*	6.5 (1.5 - 27.4)
*Citrus paradisi*	6.8 (2.9 - 15.7)
*Citrus reticulata*	7 (2.7 - 18.3)
*Citrus sinensis*	3.9 (1.2 - 13)
*Coriandrum sativum*	6.9 (3.7 - 13)
*Eucalyptus cinerea*	5.5 (4.1 - 7.4)
*Laurus nobilis*	6.2 (1.8 -21.1)
*Mentha piperita*	24.1 (5.9 - 98.7)
*Myristica fragrans*	8.8 (3 - 26)
*Pimpinella anisum*	22.4 (3.4 - 148.9)
*Syzygium aromaticum*	85.2 (2.6 - 276.8)

The insecticidal activities of the most abundant Ts in some of the EOs were compared with the insecticidal activities of the EOs themselves (Table 3). In general, the Ts were less active than the EOs. The most active T was 1,8-cineole (LC_50_ = 3.3 mg/dm^3^), followed by γ-terpinene, (4*S*)(-)-limonene, α-terpinene, and (4*R*)(+)-limonene, with LC_50_s of 4, 5, 6.2, and 6.2 mg/dm^3^, respectively. 

**Table 3:** LC_50_s of selected terpenes against *Musca domestica.*

Terpene	Mean LC_50_ in mg/dm^3^ (95% CI)
*trans*-anethole	20.5 (3.9 - 107.1)
carvacrol	45.4 (17.1 - 120.8)
citronellal	8.1 (2.8 - 23.5)
1,8-cineole	3.3 (1.1 - 10.4)
eugenol	98.4 (0.2 - 55137.1)
(4*R*)(+)-limonene	6.2 (1.7 - 23)
(4*S*)(-)-limonene	5 (2.4 - 10.4)
linalool	13.6 (0.5 - 348.6)
menthol	> 100
(±)-α-pinene	11.5 (3.6 - 37.3)
(1*R*)(+)α-pinene	12.1 (9.5 - 15.5)
(1*S*)(-)α-pinene	8.9 (2.6 - 30.8)
(1*S*)-(-)β-pinene	6.4 (2.4 - 17.4)
α-terpinene	6.2 (2.8 - 13.7)
γ-terpinene	4 (1.5 - 10.9)
terpineol	36.8 (21.1 - 63.9)
thymol	13 (2.4 - 68.7)
dimethyl 2,2-dichlorovinyl phosphate (DDVP)	0.5 (0.11-2.06)

1,8-Cineole was present in the EO from *E. cinerea*, *L. nobilis* and *M. piperita*, (in order of descending concentration). The proportion of 1,8-cineole in the *E. cinerea* EO was 56.86%, which means that the LC_50_ dose of *E. cinerea* EO contains approximately 3.14 mg of 1,8-cineole. This suggests that 1,8-cineole is the primary active component in *E. cinerea* EO. However, the presence of 1,8-cineole in other EOs does not seem to influence the magnitude of their LC_50_s greatly, since the proportion of 1,8-cineole in those EOs did not correlate (r^2^ = 0.57) with the corresponding LC_50_.

Neither δ-terpinene nor α-terpinene was a major component in any EO studied, and their presence did not seem to influence the activity of the EO that contained them. The LC_50_s of (4*S*)(-)-limonene and (4*R*)(+)-limonene were 5 and 6.2 mg/dm^3^, respectively; however, the *Citrus* EO used in this study contained only (4*R*)(+)-limonene [21,22] (Table 1). Therefore, we compared the LC_50_ of *Citrus* EOs with the LC_50_ of (4*R*)(+)-limonene and found that (4*R*)(+)-limonene, the fourth most active T, had the same insecticidal activity as *Citrus* EOs. Although its presence in citrus peel-derived EOs could help to explain the activity of *Citrus* EOs, it is remarkable that sweet orange EO, consisting of 92.47% limonene, was more active (LC_50_ = 3.9 mg/dm^3^) than (4*R*)(+)- limonene. The*C. paradisi* EO (LC_50_ = 6.8 mg/dm^3^), containing 94.97% limonene, showed a toxicity similar to that of (4*R*)(+)-limonene, but was less active than *C. sinensis* EO. Moreover, we found no correlation between the proportion of limonene in *Citrus* EO and the LC_50_ (r^2^ = 0.15). 

Dimethyl 2,2-dichlorovinyl phosphate (DDVP) selected as a positive control, showed an LC_50_ of 0.5 mg/dm^3^ (Table 3). The most active EO, *C. sinensis*, and the most active T, 1,8-cineole, were about 7.8- and 6.6-times less active than DDVP, respectively. Therefore, the EO of *C. sinensis* for example, could compete with synthetic insecticides as a fumigant in the control of *M. domestica*. The need for higher doses of these compounds does not pose a health problem because *Citrus* EOs are not highly toxic to humans, nor does it pose an environmental problem because EOs are biodegradable. 

When treated with these EOs, *M. domestica* exhibited classic signs of neurotoxicity, such as overt hyperactivity, loss of coordination, and tremors. Similar signs were described previously for exposure to pure monoterpenes [8]. Despite abundant research, the modes of action of monoterpenes or EOs are not well understood. Research has shown that some Ts have affinity for acetylcholinesterase [23] while others bind to octopamine receptors [24]. Quantitative structure-activity relationship (QSAR) studies on the insecticidal activity of thymol- and methyl-cyclohexyl-related monoterpenes against *M. domestica* demonstrated that the mode of action of these compounds might not be defined exclusively by one mechanism [25]. 

## 3. Experimental

### 3.1. General

*Trans*-Anethole, carvacrol; citronellal, 1,8-cineole; eugenol; (4*R*)(+)-limonene; (4*S*)(-)-limonene; linalool; menthol; (±)-α-pinene; (1*R*)(+)-α-pinene; (1*S*)(-)-α-pinene; (1*S*)(-)β-pinene; α-terpinene; γ-terpinene; terpineol and thymol were purchased from Sigma-Aldrich (St. Louis, MO, USA). HPLC-grade acetone was purchased from Merck (Darmstadt, Germany). Dimethyl-2,2-dichlorovinyl phosphate (DDVP) was a gift of Professor H. Masuh, from Center of Investigation on Pests and Insecticides, CONICET, Argentina. EO component analysis was performed by gas chromatography/ mass spectroscopy (GC/MS) on a Hewlett-Packard 5890 GC interfaced with a Hewlett-Packard 5970 Series mass spectrometer fitted with a HP-5MS column (15 m × 0.25 mm inner diameter). A chiral column (SUPELCO-beta-DEX 120, 60 m × 0.25 mm inner diameter) was used to resolve enantiomers.

### 3.2. Plant material

Twelve aromatic edible plants or fruits were selected: *Citrus aurantium* L. (bitter orange) fruit peel, *Citrus limon* L. (lemon) fruit peel, *Citrus paradisi* Macf. (grapefruit) fruit peel, *Citrus reticulata* Blanco (mandarin) fruit peel, *Citrus sinensis* L. (sweet orange) fruit peel*, Coriandrum sativum* L. (coriandrum) seeds, *Eucalyptus cinerea* F. v. Muell.(eucalyptus) leaves, *Laurus nobilis* L. (bay) leaves, *Mentha piperita* L. (peppermint) leaves, *Myristica fragrans* Houtt. (nutmeg) nut, *Pimpinella anisum* L. (anise) seeds and *Syzygium aromaticum* (L.) Merr. & Perry. (cloves) dried flower buds. The fruits and leaves were obtained from domestic organic fruit gardens. Seeds and nuts were purchased from local grocery stores.

### 3.3. Essential oil extraction and analysis

Essential oils were extracted for 2 h by hydrodistillation in a Clevenger-type apparatus with a separated extraction chamber. The EOs were dried over anhydrous sodium sulfate. EO component analysis was performed by gas chromatography/mass spectroscopy (GC/MS) using the instrumentation described above. The temperature program was 50° to 240°C at 5°C/min. Helium was used as the carrier gas (flow rate = 0.9 mL/min). Chiral analyses were performed with the same temperature program. The mass spectra were obtained at an ionization voltage of 70 eV. The identification of compounds in the chromatographic profiles was achieved by comparison of their mass spectra with a library database (NIST, version 3.0), and was confirmed by comparison of retention indices with those of authentic standards (terpenes listed in Section 3.1) or with values from the literature. The percentage composition of the oils was computed by area normalization method from GC peak areas calculated as the mean value of two injections from each oil.

### 3.4. Fly collection and maintenance

Colonies of *M. domestica* originated from adults were collected from the experimental field of the Universidad Católica of Córdoba, in Córdoba, Argentina, using a sweep net. The flies were transferred to a small cage and then reared in entomological cages (30×30×30 cm) at 26 (±1) °C under a 12:12 light:dark cycle and 70% humidity. Adult flies were provided with water and fed a 1:1 (v/v; approximately) mixture of granulated sugar and powdered milk. Bran and milk were prepared at a weight ratio of 1:3 and 100 g of this mixture was placed on a plastic plate as an oviposition site. 

### 3.5. Bioassay

The bioassay was designed such that the flies would have high probability of coming into contact with the volatile compounds within the 30 minute test period; therefore, the flies were allowed access to the total space within the exposure vessel. Ten 4-5 day-old adult house flies, mixed sexes, were placed in a glass jar (1.2 dm^3^) fitted with a screw cap with a 7-cm length of cotton yarn suspended from the center of the internal face of the cap. Different dosages of pure EO (without solvent) were applied to the yarn. The control vessel had no compound on the cotton yarn. In cases of Ts that are solids at room temperature, such as menthol and thymol, each dose was applied after being dissolved in 10 μL of acetone; in these cases, the cotton yarn in the control vessel was treated with 10 μL of acetone. The jars were then sealed tightly. The jars were maintained in a room at 26 ± 1 °C. Each test was replicated three times. The assay was also run with the cotton yarn enclosed in a bag made of breathable cloth so that the flies could not contact the yarn. Dimethyl 2, 2-dichlorovinyl phosphate (DDVP), a volatile organophosphate, was used as a positive control. Mortality in each group was assessed after 30 min of exposure by softly stimulating each fly with the tip of a pen. Flies that did not respond were considered dead. The mean mortality data of the three replicates per doses (4-6 doses per EO or compound) was used to calculate the LC_50_. Probit analysis (Harvard Programming; Hg1, 2) was used to analyze the dose-mortality response.

## 4. Conclusions

The above mentioned results indicate that: a) The EOs from *C. sinensis*, *C. aurantium*, and *E. cinerea* were highly toxic to adult houseflies, provoking death within a short period of time; b) in many cases, the EOs are more effective, as housefly fumigants, than their most abundant T component; c) the most active EO of this study, *C. sinensis* EO, showed in 30 min experiment a comparable LC_50_ (= 3.9 mg/dm^3^) to that determined by Pavela *et al*. for *Mentha pulegium* (LC_50_= 4.7 mg/dm^3^) at 24 h [20]; and d) EOs from *C. sinensis*, *C. aurantium*, and *E. cinerea* are highly effective and may be considered good candidates for development as fumigants that are compatible with food, property, and human habitats.
